# FUT8 promotes breast cancer cell invasiveness by remodeling TGF-β receptor core fucosylation

**DOI:** 10.1186/s13058-017-0904-8

**Published:** 2017-10-05

**Authors:** Cheng-Fen Tu, Meng-Ying Wu, Yuh-Charn Lin, Reiji Kannagi, Ruey-Bing Yang

**Affiliations:** 1 Institute of Biomedical Sciences, Academia Sinica, Taipei, 11529 Taiwan; 2 Institute of Pharmacology, National Yang-Ming University, Taipei, 11221 Taiwan; 3 Ph.D. Program in Biotechnology Research and Development, College of Pharmacy, Taipei Medical University, Taipei, 11031 Taiwan

**Keywords:** Breast cancer, Core fucosylation, EMT, FUT8, Metastasis, TGF-β receptor

## Abstract

**Background:**

Core fucosylation (addition of fucose in α-1,6-linkage to core N-acetylglucosamine of N-glycans) catalyzed by fucosyltransferase 8 (FUT8) is critical for signaling receptors involved in many physiological and pathological processes such as cell growth, adhesion, and tumor metastasis. Transforming growth factor-β (TGF-β)-induced epithelial–mesenchymal transition (EMT) regulates the invasion and metastasis of breast tumors. However, whether receptor core fucosylation affects TGF-β signaling during breast cancer progression remains largely unknown.

**Method:**

In this study, gene expression profiling and western blot were used to validate the EMT-associated expression of FUT8. Lentivirus-mediated gain-of-function study, short hairpin RNA (shRNA) or CRISPR/Cas9-mediated loss-of-function studies and pharmacological inhibition of FUT8 were used to elucidate the molecular function of FUT8 during TGF-β-induced EMT in breast carcinoma cells. In addition, lectin blot, luciferase assay, and in vitro ligand binding assay were employed to demonstrate the involvement of FUT8 in the TGF-β1 signaling pathway. The role of FUT8 in breast cancer migration, invasion, and metastasis was confirmed using an in vitro transwell assay and mammary fat pad xenograft in vivo tumor model.

**Results:**

Gene expression profiling analysis revealed that FUT8 is upregulated in TGF-β-induced EMT; the process was associated with the migratory and invasive abilities of several breast carcinoma cell lines. Gain-of-function and loss-of-function studies demonstrated that FUT8 overexpression stimulated the EMT process, whereas FUT8 knockdown suppressed the invasiveness of highly aggressive breast carcinoma cells. Furthermore, TGF-β receptor complexes might be core fucosylated by FUT8 to facilitate TGF-β binding and enhance downstream signaling. Importantly, FUT8 inhibition suppressed the invasive ability of highly metastatic breast cancer cells and impaired their lung metastasis.

**Conclusions:**

Our results reveal a positive feedback mechanism of FUT8-mediated receptor core fucosylation that promotes TGF-β signaling and EMT, thus stimulating breast cancer cell invasion and metastasis.

**Electronic supplementary material:**

The online version of this article (doi:10.1186/s13058-017-0904-8) contains supplementary material, which is available to authorized users.

## Background

Breast cancer is the most common malignant tumor and second only to lung and bronchus cancer as the cause of cancer-related deaths in women, because it often leads to metastatic disease [1]. The spread of tumor cells from a primary tumor to a secondary site remains one of the most life-threatening pathological events.

During the cellular epithelial–mesenchymal transition (EMT), epithelial tumor cells lose their polarized organization and cell–cell junctions. Cells undergo changes in shape and cytoskeletal organization and acquire mesenchymal characteristics important for metastasis [2, 3]. Transforming growth factor-β (TGF-β)-induced EMT regulates the invasion and metastasis of breast tumors [4, 5]. Dissecting the molecular mechanisms that regulate EMT is pivotal for controlling tumor invasiveness and metastasis.

Glycosylation is the stepwise procedure of covalent attachment of oligosaccharide chains to proteins or lipids. The carbohydrate structures of cell-surface glycoconjugates play an important role in many physiological and pathological events, including cell growth, differentiation, and transformation [6]. Fucosylation is one of the most important types of glycosylation in cancer. Altered fucosylation has been implicated in malignant transformation, invasion, and metastasis of certain types of cancer, such as hepatocellular, gastric, pancreatic, prostate, and colorectal cancers [7–11].

To date, 13 different fucosyltransferases (FUTs) have been identified in the human genome [12]. FUT8 is the only fucosyltransferase involved in core fucosylation (addition of fucose in α-1,6-linkage to the innermost N-acetyl glucosamine of N-glycans) [13]. Core fucosylation of glycoproteins has important regulatory functions for adhesion molecules and growth factor receptors, such as α3β1 integrin, epidermal growth factor receptor, and hepatocyte growth factor receptor [14–16]. Recently, FUT8 expression was found to be associated with poor prognosis in breast cancer [17]. However, the exact molecular function of FUT8 during breast cancer progression and whether FUT8 could be a potential therapeutic target remains largely unknown.

In this study, we identified that FUT8 is highly upregulated in TGFβ-induced EMT and associated with the migratory and invasive ability of aggressive breast carcinoma cell lines. In addition, gain-of-function and loss-of-function studies have demonstrated that FUT8 overexpression stimulates EMT, whereas FUT8 knockdown suppresses the invasiveness of highly aggressive breast carcinoma cells. Furthermore, the heteromeric complexes of TGF-β receptors I and II (RI and RII) were direct targets of FUT8, and their core fucosylation markedly facilitated ligand binding and promoted TGF-β signaling activity. Importantly, pharmacological inhibition by a small molecule inhibitor, 2-fluorinated-peracetyl-fucose (2 F-peracetyl-fucose), or genetic inactivation of FUT8 suppressed the invasive ability of aggressive breast cancer cells in vitro and impaired their lung metastasis in vivo. Our results unravel a novel feed-forward mechanism of FUT8-mediated TGF-β receptor core fucosylation that stimulates breast cancer cell invasion and metastasis. Targeting FUT8 could be a potential therapeutic strategy for breast cancer treatment.

## Methods

### Cell culture

MCF-10A, MDA-MB-231, Hs578T, T-47D cell lines were obtained from the American Type Culture Collection (Manassas, VA, USA). Mammary epithelial MCF-10A cells were maintained in DMEM/F-12 with 10 ng/ml insulin, 100 ng/ml cholera toxin, 500 ng/ml hydrocortisone, 20 ng/ml epidermal growth factor, and 5% goat serum. Human breast cancer MDA-MB-231 and Hs578T cells were propagated in DMEM with 10% fetal bovine serum (FBS). Human breast cancer T-47D cells and murine breast cancer 4T1 cells were propagated in RPMI 1640 with 10% FBS. Three-dimensional (3D) laminin-rich extracellular matrix (3D lrECM) on-top cultures [18] were prepared by trypsinization of cells from tissue culture plastic, seeding of single cells on a thin layer of Matrigel (Corning Inc., Corning, NY, USA) and addition of medium containing 5% of Engelbreth-Holm-Swarm (EHS) extracellular matrix extracts. To induce EMT in 3D cultured cells, culture medium was supplemented with 10 ng/ml TGF-β1 (R&D systems, Minneapolis, MN, USA) for 8 days to generate mesenchymal-like cells.

### Immunoprecipitation, western blot analysis, and lectin blot analysis

Two days after transfection, cell lysates were clarified by centrifugation at 10,000 × g for 20 min at 4 °C. Samples were incubated with 1 μg of indicated antibody and 20 μl of 50% (v/v) Protein A-agarose (Pierce) for 2 h with gentle rocking. After three washes with lysis buffer, precipitated complexes were solubilized by boiling in Laemmli sample buffer, fractionated by SDS-PAGE, and transferred onto polyvinylidene fluoride (PVDF) membranes, which were blocked with phosphate buffered saline (PBS; pH 7.5) containing 0.1% gelatin and 0.05% Tween 20 and blotted with the indicated antibodies. After two washes, blots were incubated with horseradish peroxidase (HRP)-conjugated goat anti-mouse/rabbit IgG (Jackson ImmunoResearch Laboratories) for 1 h. After washing, reactive bands were visualized using the VisGlow chemiluminescent substrate, HRP system (Visual Protein, Taipei). For lectin blot analysis, membranes were blocked with PBS (pH 7.5) containing 0.1% gelatin and 0.05% Tween 20, detected with biotinylated *Lens culinaris* lectin (LCA) (Vector Laboratories, Burlingame, CA, USA), then incubated with HRP-conjugated streptavidin (Vector Laboratories).

### Flow cytometry

Cells were collected and suspended in PBS/ 2% FBS in a volume of 0.5 ml. Cell suspensions were incubated with fluorescein-labeled LCA (Vector Laboratories) on ice for 1 h. After washing three times with ice-cold PBS, the cells were resuspended in 0.5 ml of PBS/2% FBS. Flow cytometry involved use of FACSCalibur (BD Biosciences, San Jose, CA, USA).

### CRISPR/Cas9-mediated genome editing

To generate *FUT8*-knockout (FUT8-KO) cells with the CRISPR/Cas9 system, guide RNAs (gRNAs) targeting the human *FUT8* gene at exon 3 or 6 were cloned into the GeneArt CRISPR Nuclease Vector (Thermo Fisher Scientific, Waltham, MA, USA). After sequence verification of the insert, the CRISPR/Cas9 plasmids were transfected into HEK-293 T or MDA-MB-231 cells. Two days after transfection, cells underwent flow cytometry-based sorting of crRNA (CRISPR RNA)-expressing cell populations with orange fluorescent protein (OFP) expression. These crRNA-expressing cell populations were further cultured for 1 week, and FUT8-KO cells were selected by fluorescence-activated cell sorting (FACS) analysis with LCA binding. Genomic indel modification of FUT8 in single-cell clones was assessed by PCR and sequencing.

### RNA extraction, complementary DNA (cDNA) synthesis, and RT-PCR

Total RNA was prepared from cultured cells by the TRIzol method (Thermo Fisher Scientific, Waltham, MA, USA). First-strand cDNA synthesis with SuperScript II reverse transcriptase (Thermo Fisher Scientific) involved 5 μg RNA. The first-strand cDNA reaction was used for each PCR as a template.

### Cell migration and invasion assay

Cell migration and invasion was measured in a Boyden chamber system according to standard protocols [19]. MDA-MB-231 and 4T1 cells were cultured in serum-free medium for 24 h. For migration assays, cells (1 × 10^5^) were placed in the upper chamber with non-coated membrane (24-well insert; 8-μm pore size; Corning Inc.). For invasion assays, cells (1 × 10^5^) were placed in the top chamber with Matrigel-coated membrane (24-well insert; 8-μm pore size; Corning Inc.) In both assays, cells were plated in 0.2 ml serum-free medium in the top chamber, and the lower chamber was loaded with 0.5 ml medium containing 10% FBS. The total number of cells that migrated into the lower chamber was counted after 16 h of incubation at 37 °C with 5% CO_2_. Cells that had not penetrated the filter were wiped out with cotton swabs, and cells that had migrated to the lower surface of the filter were stained with 0.5% crystal violet, examined by bright field microscopy, and photographed. Crystal violet was then dissolved with ethanol, and absorbance was read at 570 nm. Values for migration/invasion were expressed as the average number of optical density (OD)_570_ per assay.

### Xenograft breast cancer mouse model

Female athymic mice (8-week-old nu/nu strain BALB/cAnN.Cg-Foxn1nu/CrlNarl) were implanted with control or FUT8-kncodown 4T1 cells (2 × 10^5^ cells) in the mammary fat pads. Tumor growth was monitored weekly by measuring perpendicular tumor diameters, length (L) and width (W), with a Vernier caliper. The tumor volume (V) was calculated as V = LW^2^/2. Mice were sacrificed 4 weeks after tumor implantation. Metastatic nodules in the lungs were quantified under a dissecting microscope, then the lungs were fixed, embedded, sectioned, and stained with hematoxylin and eosin (H&E). Inhibition of fucosylation using 2 F-Peracetyl-Fucose (Millipore Calbiochem, Darmstadt, Germany) was described by Rillahan et al. [20]. The 4T1 cells were pretreated with 300 μM 2 F-Peracetyl-Fucose or dimethyl sulfoxide (DMSO) for 7 days before xenograft. After tumor implantation, mice were administered with 2 F-peracetyl-fucose or vehicle control three times per week by oral gavage.

### Luciferase activity assay

MDA-MB-231 cells (2 × 10^5^ cells per well) were seeded in 24-well plates and transfected with 0.3 μg of the TGF-responsive luciferase reporter 3TP-lux, with 0.01 μg Renilla luciferase reporter as an internal control. Transfected cells were maintained in low-serum medium (0.1% FBS) for 18 h. Luciferase activity was measured after 24-h treatment with recombinant human TGF-β1 protein (R&D systems).

### Indirect TGF-β1 binding assay

The HIS-tagged TGF-β1 (HIS.TGF-β1) ligand was constructed by amplifying the TGF-β1 cDNA by PCR with the primer sequences AAG CTT GCT ATC CAC CTG CAA GAC TAT and CTC GAG CGC TGC ACT TGC AGG AGC GCA. The amplified fragment was then cloned into the HindIII and XhoI restriction sites of pSecTag2 vector (Invitrogen). HIS.TGF-β1 protein was produced by transient transfection into HEK-293 T cells. After 2 days, conditioned medium was collected and added to control or FUT8-KO MDA-MB-231 cells endogenously expressing TGF-β receptors for 4 h at 4 °C. After stringent washing, bound HIS.TGF-β1 protein was detected with AP (alkaline phosphatase)-conjugated anti-HIS antibody. After washing, cells were lysed for 10 min on ice in lysis buffer (25 mM Hepes, pH 7.6, 150 mM NaCl, 5 mM EDTA, 10 μg/ml aprotinin, 5 μg/ml leupeptin, 10% glycerol, and 1% Triton X-100). Cell lysates were clarified by centrifugation at 10,000 × g for 15 min at 4 °C. The bound AP protein was reacted with p-nitrophenyl phosphate substrate (Sigma) to quantify AP binding in cell extracts.

### Statistical analysis

Data are expressed as mean ± SD and were analyzed by paired *t* test. *P* < 0.05 was considered statistically significant. The relationship between *FUT8* expression and survival of breast cancer patients was evaluated by Kaplan-Meier survival analysis with the PrognoScan database [21].

## Results

### FUT8 is upregulated during TGF-β-induced EMT

Extracellular matrix is a key regulator of normal homeostasis and tissue phenotype. Important signals are lost when cells are cultured ex vivo on 2D plastic substrata. Many of these crucial micro-environmental cues may be restored by using 3D cultures of laminin-rich extracellular matrix [22]. Thus, appropriate 3D culture provides a more physiologically or pathologically relevant approach to dissect gene functions and cellular phenotypes ex vivo.

**Fig. 1:**
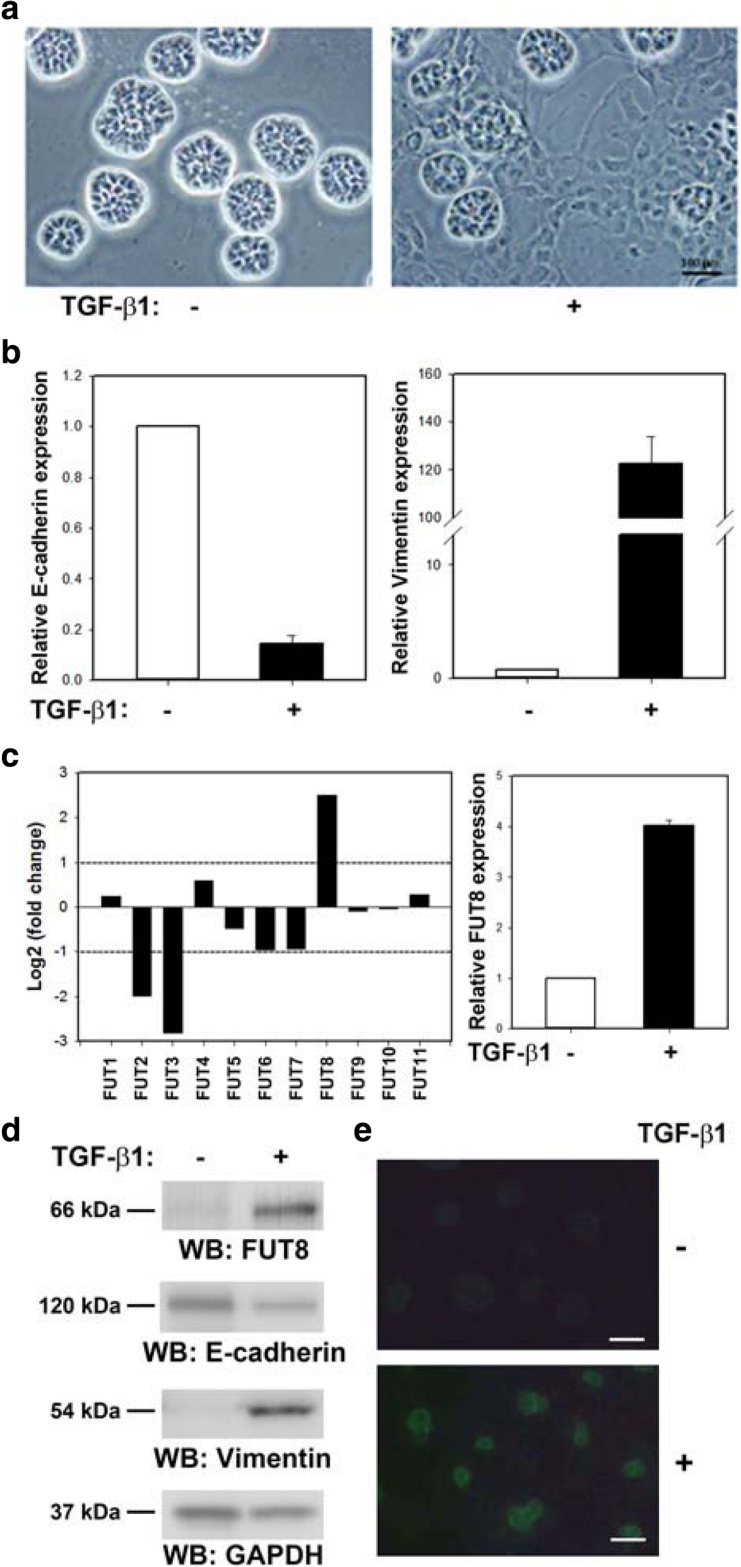
Fucosyltransferase 8 (FUT8) expression is upregulated and core fucosylation is increased during epithelial-mesenchymal transition (EMT) in MCF-10A cells. **a**-**b** Transforming growth factor-β (TGF-β)-induced EMT in a 3D culture system. After TGF-β1 treatment for 8 days, TGF-β-induced EMT was characterized by morphological transformation of polarized, acini-like spheroids into a fibroblastic cell shape (**a**) and verified molecularly by E-cadherin downregulation and vimentin upregulation (**b**). **c** *FUT8* is the only *FUT* gene highly upregulated during TGF-β1-stimulated EMT. Gene expression profiling data on *FUT* genes presented as log2-fold ratio difference between TGF-β-induced EMT and control MCF-10A cells (left panel). Quantitative PCR analysis of *FUT8* mRNA expression before and after TGF-β-induced EMT (right panel). **d** Western blot analysis of FUT8, E-cadherin, and vimentin protein expression after TGF-β-induced EMT. Glyceraldehyde-3-phosphate dehydrogenase (GAPDH) was a loading control. **e** *Lens culinaris* agglutinin immunofluorescence staining (carbohydrate probe for core fucose) of cell-surface core fucosylation during EMT. Scale bar = 50 μm. Data are mean ± SE. kDa, kiloDalton

We first reproduced a TGF-β-induced EMT model in a 3D culture system as described [18] in the MCF-10A human breast epithelial cell line (Fig. 1a and b), and used this 3D-EMT model to examine the potential connection between FUT8 and EMT. TGF-β-induced EMT was confirmed by morphological disruption of the polarized, acini-like spheroids recapitulating the glandular architecture in vivo and verified molecularly by quantitative RT-PCR (Fig. 1b) and western blot analysis (Fig. 1d) showing E-cadherin downregulation and vimentin upregulation. Gene expression analysis revealed that among all 11 *FUT* gene members, *FUT8* was the only highly expressed FUT during TGF-β-induced EMT in breast epithelial MCF-10A cells (Fig. 1c, left panel). In line with this finding, quantitative RT-PCR analysis confirmed upregulated *FUT8* during EMT (Fig. 1c, right panel). In addition, western blot analysis validated markedly increased FUT8 protein expression after EMT (Fig. 1d).

LCA, a plant lectin, can selectively recognize the core fucose of N-glycan [13], so we examined whether global core fucosylation occurs during EMT by using fluorescein-labeled LCA. LCA-FITC staining was significantly increased with TGF-β-stimulated EMT in MCF-10A cells (Fig. 1e). Hence, FUT8 induction is concomitant with an overall increase in core fucosylation on the surface of human breast epithelial cells during TGF-β–induced EMT.

### FUT8 is critical for TGF-β-induced EMT in MCF-10A cells

**Fig. 2:**
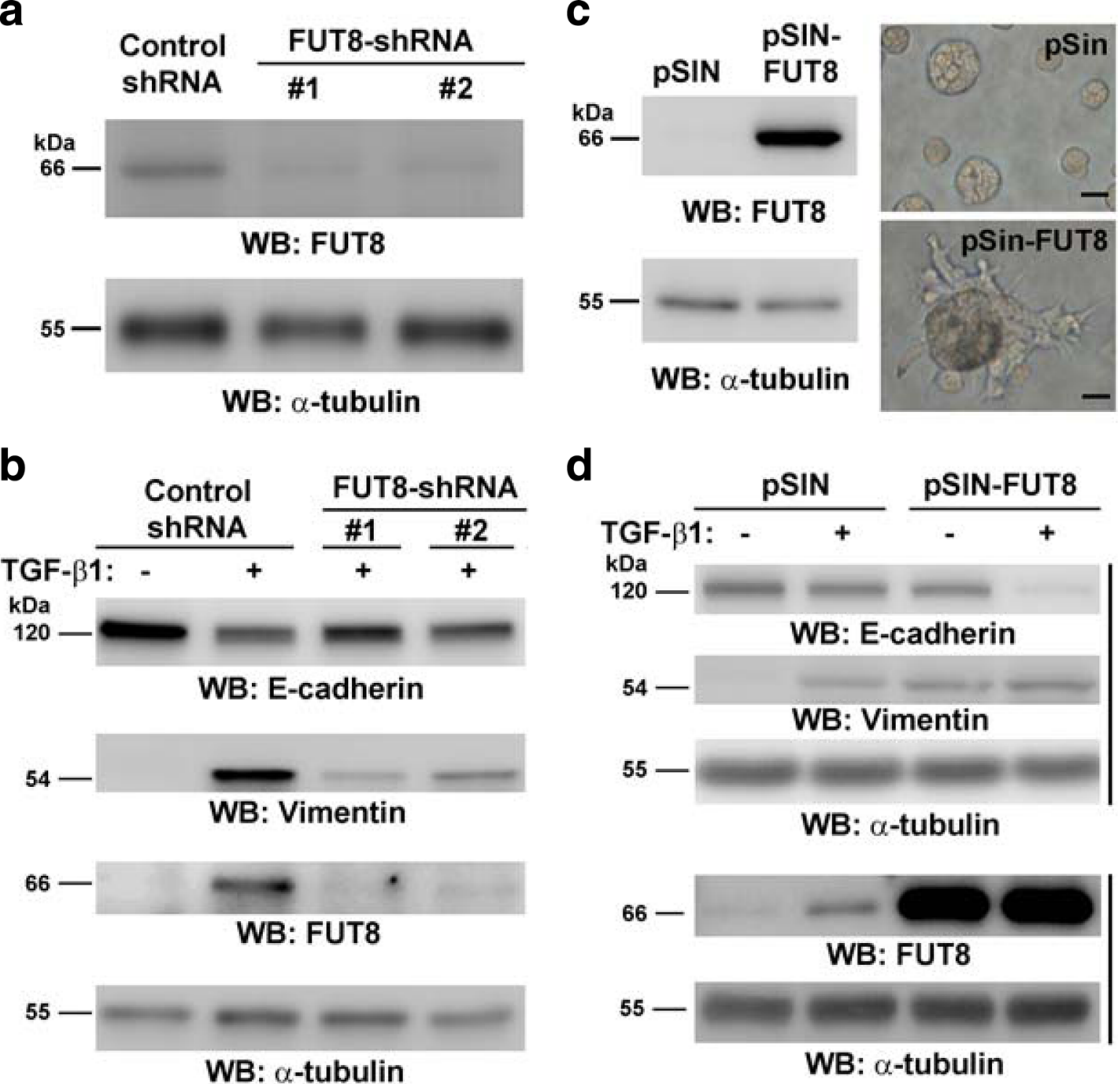
Fucosyltransferase 8 (FUT8) plays a critical role in transforming growth factor-β1 (TGF-β1)-induced epithelial–mesenchymal transition (EMT). **a** Knockdown of FUT8 expression in MCF-10A cells. MCF-10A cells were infected with a lentiviral vector to generate a stable clone expressing a short hairpin RNA (shRNA) control or two independent shRNAs targeting FUT8 (FUT8-shRNA #1 and #2), respectively. The efficiency of FUT8 knockdown in the selected stable pool was confirmed by western blot analysis. α-Tubulin expression was an internal control. **b** FUT8 knockdown impaired the EMT in MCF-10A cells. Control or FUT8-knockdown MCF-10A cells were treated with TGF-β1 (10 ng/ml) for 8 days. The effect of FUT8 knockdown on the EMT was examined by measuring E-cadherin and vimentin expression by western blot analysis as previously described. **c** Overexpression of FUT8 produces invasive, nonpolar, and disorganized acini in MCF-10A cells. MCF-10A cells were infected with a lentiviral vector to generate a stable clone expressing control (pSin) or FUT8 (pSIN-FUT8). FUT8 overexpression was confirmed by western blot analysis (left panel). FUT8-overexpressing MCF-10A cells show invasive and disorganized acini on 3D culture (right panel). **d** Overexpression of FUT8 promoted EMT-like phenotype in MCF-10A cells. Control or FUT8-overexpressing MCF-10A cells were treated with TGF-β1 for 8 days. The EMT was determined by measuring the E-cadherin and vimentin expression (top panel). FUT8 expression was examined by western blot analysis (bottom panel). α-Tubulin expression was examined as internal control individually. kDa, kiloDalton

To investigate the involvement of FUT8 during EMT, we knocked down or overexpressed FUT8 in MCF-10A cells and examined the effects on EMT by determining the E-cadherin and vimentin expression switch. Silencing FUT8 expression with two independent short hairpin RNAs (shRNAs) (FUT8-shRNA #1 and #2), as confirmed by western blot analysis (Fig. 2a), impaired TGF-β-stimulated EMT: E-cadherin expression remained high and vimentin expression low in MCF-10A cells (Fig. 2b). Similarly, the effect of FUT8 knockdown that significantly impaired TGF-β-induced EMT was confirmed in one additional mammary glandular epithelial NMuMG cell line (Additional file 1: Figure S1). Lentivirus-mediated FUT8 overexpression (pSIN-FUT8) alone in MCF-10A cells, as confirmed by western blot analysis (Fig. 2c), was sufficient to support the formation of invasive filopodia, generate non-polarized acini and significantly facilitate TGF-β-induced EMT in MCF-10A cells (Fig. 2c and d). These results indicated that FUT8 plays an important role in promoting the EMT in human breast epithelial MCF-10A cells.

### FUT8 level is positively associated with invasion ability of breast cancer cell lines

**Fig. 3:**
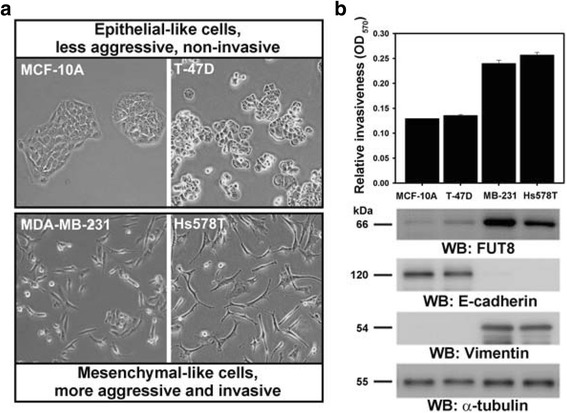
Increased fucosyltransferase 8 (FUT8) expression is associated with the invasive ability of breast cancer cell lines. **a** Cell morphology of normal human epithelial cells (MCF-10A), low-metastatic breast cancer cells (T-47D) and highly invasive breast cancer cells (MDA-MB-231 and Hs578T). Of note, on Transwell assay, the former two cell lines were non-invasive and had an epithelium-like morphology (upper), whereas the latter two were highly invasive and had a spindle-shaped, fibroblastic mesenchymal phenotype (lower). **b** Western blot analysis of FUT8 protein level in cells. The invasion ability (upper panel) of breast carcinoma cell lines was determined by Transwell invasion assay. Data are mean ± SD. OD, optical density

To investigate whether FUT8 is functionally involved in regulating the aggressiveness of breast cancer cells, we examined the expression of FUT8 in epithelial-like normal human epithelial cells (MCF-10A), low-metastatic breast cancer cells (T-47D) and mesenchymal-like highly invasive breast cancer cell lines (MDA-MB-231 and Hs578T) (Fig. 3a). FUT8 protein level was higher in highly invasive breast cancer cells (MDA-MB-231 and Hs578T) than normal human epithelial cells and low-metastatic breast cancer cells (MCF-10A and T-47D) (Fig. 3b). Our mining data reveal a trend toward elevated *FUT8* expression at mRNA levels in distal metastatic tumors compared to primary breast tumors or in invasive ductal carcinoma (IDC) compared to non-invasive ductal carcinoma in situ (DCIS) (Additional file 2: Figure S2). In agreement with this expression profile, in silico Kaplan-Meier survival analysis of three public microarray datasets [23–25] revealed that high *FUT8* levels were associated with poor prognosis in breast cancer (Additional file 3: Figure S3). Together, these findings imply that FUT8 upregulation is associated with cancer cell invasiveness and poor prognosis in breast cancer, which agrees with a recent report [17].

### FUT8 knockdown suppresses migration and invasiveness of MDA-MB-231 and 4T1 breast carcinoma cells

**Fig. 4:**
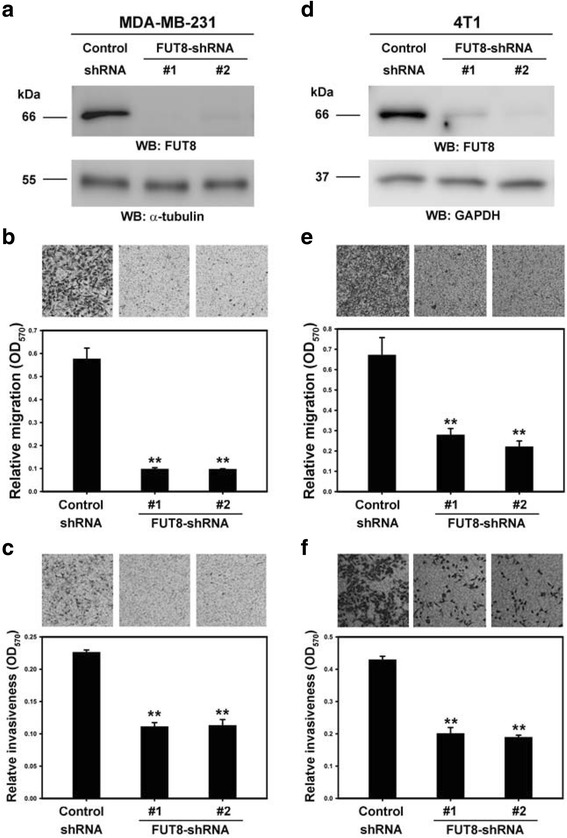
Knockdown of fucosyltransferase 8 (FUT8) suppresses the invasion ability of two highly metastatic breast carcinoma cell lines. Knockdown of FUT8 expression with recombinant lentivirus encoding two different human FUT8-targeting shRNAs and murine FUT8-targeting short hairpin RNAs (shRNAs) in MDA-MB-231 (**a**-**c**) and 4T1 cells (**d**-**f**). Knockdown efficiency was confirmed by western blot analysis. Anti-α-tubulin and anti- glyceraldehyde-3-phosphate dehydrogenase (GAPDH) antibodies were internal controls. Cell migratory (**b**, **e**) and invasive ability (**c**, **f**) of control and FUT8-knockdown MDA-MB-231 and 4T1 cells by Transwell assay with and without Matrigel coating. Data are mean ± SD. **P < 0.01. OD, optical density; kDa, kiloDalton

Cell migration and invasion are critical steps in tumor progression and metastasis. We used the highly invasive breast carcinoma cell lines, MDA-MB-231 and 4T1 cells, to determine the biological function of FUT8 in cell migration and invasion. FUT8 expression was knocked down by the two different FUT8-targeting shRNAs in MDA-MB-231 or 4T1 cells (Fig. 4a and d) and examined for an effect on cell migration and invasiveness in vitro. FUT8 knockdown significantly reduced the migratory and invasive abilities of MDA-MB-231 (Fig. 4b and c) and 4T1 cells (Fig. 4e and f).

### TGF-β receptor proteins I and II (RI and RII) are modified by core fucosylation dependent on FUT8

**Fig. 5:**
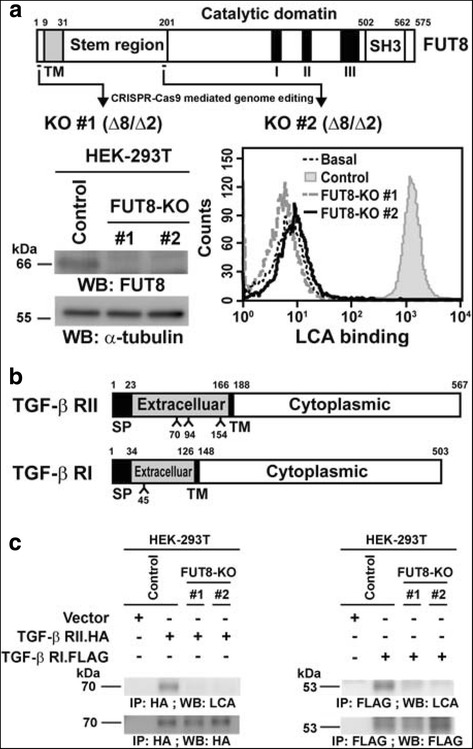
Transforming growth factor-β (TGF-β) receptor proteins I and II (RI and RII) are core fucosylated by fucosyltransferase 8 (FUT8) in HEK-293 T cells. **a** Establishment of FUT8-knockout (KO) HEK-293 T cells by CRISPR-Cas9-mediated genome editing. Two independent CRISPR-Cas9 clones targeting exon 3 or 6 of *FUT8* were established (upper panel). Inactivation of *FUT8* gene and consequent loss of core fucosylation were validated by western blot (lower-left panel) and LCA binding assay (lower-right panel). **b** Protein domain organization of TGF-β RI and RII. According to the Uniprot database (http://www.uniprot.org), potential N-linked glycosylation sites within TGF-β RII (Asn-70, 94, and 154) and RI (Asn-45) were marked. SP, signal peptide sequence; TM, transmembrane domain. **c** Core fucosylation of TGF-β RI and RII proteins were eliminated by FUT8 knockout. Recombinant TGF-β RI or RII proteins in the control or two FUT8-KO 293 T cell lines were probed with biotinylated *Lens culinaris* lectin (LCA), then detection with streptavidin-conjugated horseradish peroxidase. kDa, kiloDalton

In light of increased FUT8-mediated core fucosylation during EMT in MCF-10A cells, we determined which membrane proteins are the direct targets involved in upstream EMT signaling. TGF-β signal transduction occurs when ligands interact with heteromeric complexes of TGF-β RI and RII receptors to activate downstream transcription factors [26]. Because both receptors are N-glycoproteins with potential N-linked glycosites at Asn-70, 94, and 154 of TGF-β RII and at Asn-45 of TGF-β RI (see Fig. 5b) and because the TGF-β sensitivity can be regulated by N-linked glycosylation of TGF-β receptors [27, 28], we examined whether these two TGF-β receptor glycoproteins are modified by FUT8-catalyzed core fucosylation by using LCA lectin blot analysis.

We first generated two independent FUT8-knockout (KO) HEK-293 T cells by using the CRISPR-Cas9 system. CRISPR/Cas9-mediated genome editing is useful for targeting an individual gene and introducing indel mutations that result in in-frame amino acid deletions, insertions, or frameshift mutations leading to premature stop codons within the open reading frame of the targeted gene [29, 30]. We selected two independent CRISPR sites targeting exons 3 and 6 of FUT8 gene (see Fig. 5a). After transfecting the FUT8-targeting CRISPR/Cas9 plasmids into HEK-293 T cells, individual FUT8-KO cell clones were isolated by cell sorting, and the CRISPR/Cas9-mediated indel mutations were verified at both the genomic DNA and messenger RNA (mRNA) levels (Fig. 5a, upper panel), as was the protein level by western blot analysis (Fig. 5a, lower-left panel) and the consequent loss of core fucosylation at the cell surface by LCA binding assay (Fig. 5a, lower-right panel).

**Fig. 6:**
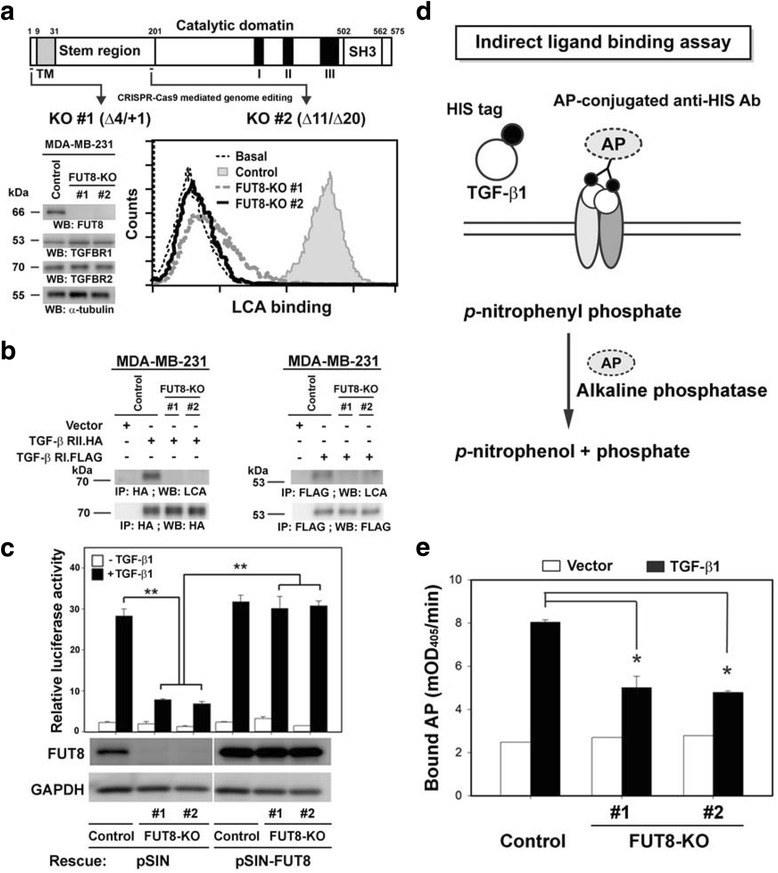
Fucosyltransferase 8 (FUT8) knockout impairs transforming growth factor-β1 (TGF) ligand binding and decreases TGF-β downstream signaling in MDA-MB-231 cells. **a** Establishment of FUT8-knockout (KO) MDA-MB-231 cells by CRISPR-Cas9-mediated genome editing. Two independent KO #1 and #2 clones targeting exon 3 or 6 of FUT8 were established (upper panel). The deletion of FUT8 and loss of core fucosylation were confirmed by western blot (lower-left panel) and LCA binding assay (lower-right panel) in MDA-MB-231 cells. Of note, FUT8 inactivation did not affect the expression of TGF-β RI or RII protein (lower-left panel). **b** Core fucosylation of TGF-β RI and RII protein were eliminated by FUT8 KO. Recombinant TGF-β RI and RII proteins produced in the control or two FUT8-KO MDA-MB-231 cell lines were probed with biotinylated *Lens culinaris* lectin (LCA), then detected by streptavidin-conjugated horseradish peroxidase. **c** Reporter assay with a TGF-β1-responsive luciferase reporter, 3TP-lux, showed significantly reduced TGF-β1-mediated signaling in FUT8-KO cells, which was rescued completely by FUT8 overexpression. **d** Schematic figure showing the indirect ligand binding assay. HEK-293 T cells were transfected with empty vector or the expression plasmid encoding HIS-tagged TGF-β1 (HIS.TGF-β1) protein. After 2 days, conditioned medium were added to MDA-MB-231 cells endogenously expressing TGF-β receptors. Bound HIS.TGF-β1 protein was quantified by the alkaline phosphatase (AP)-conjugated anti-HIS antibody reacting with its colorimetric substrate p-nitrophenyl phosphate. **e** FUT8 knockout reduced TGF-β1 ligand binding. Data are mean ± SD. *P < 0.05, **P < 0.01. mOD405, milli-absorbance units at 405 nm

When overexpressed in control HEK-293 T cells, TGF-β RI and RII proteins were indeed core fucosylated, which was completely abolished in FUT8-deficient HEK-293 T cell lines (Fig. 5c). Together, our results validated that heteromeric signaling receptors for TGF-β are true targets of FUT8 and are modified by core fucosylation, at least in HEK-293 T cells. Similar experiments were repeated in MDA-MD-231 cells. Two independent FUT8-KO MDA-MB-231 clones were generated by use of the CRISPR-Cas9 system. Inactivation of FUT8 and resulting loss of core fucosylation were verified at genomic DNA, mRNA, and protein levels by western blot and LCA binding assays (Fig. 6a). Furthermore, we confirmed that TGF-β RI and RII are indeed FUT8 target proteins and can be core-fucosylated in aggressive breast carcinoma cells because FUT8 knockout completely eliminated its core fucosylation (Fig. 6b).

### FUT8 knockdown inhibits TGF-β1 signaling and ligand binding in MDA-MB-231 cells

Because core fucosylation may affect cell-surface protein functions including receptor signaling [14–16], we further investigated whether blocking core fucosylation of TGF-β receptors by FUT8 knockdown could affect the TGF-β1 signaling pathway in aggressive breast cancer MDA-MB-231 cells. To this end, we evaluated whether FUT8 inactivation affected the TGF-β1-mediated transcriptional activity by luciferase reporter assay with a TGF-β-responsive 3TP-lux reporter [31]. The 3TP-lux reporter plasmid was transfected into control or FUT8-KO MDA-MB-231 cells, which were then stimulated with TGF-β1 (10 ng/ml) or left untreated for 24 h. TGF-β1 treatment increased the basal level of 3TP-lux reporter transcription 10-fold in control MDA-MB-231 cells, and FUT8-KO markedly diminished TGF-β1-mediated transcriptional activity (Fig. 6c). This suppressive TGF-β1 signaling activity could be fully rescued by overexpression of FUT8 by a recombinant lentiviral vector (Fig. 6c), which demonstrates the critical and specific involvement of FUT8 in TGF-β1 activity in breast cancer MDA-MB-231 cells.

To examine whether core fucosylation of TGF-β receptors is vital for its ligand binding, we further developed an indirect TGF-β ligand binding assay in which we detected the binding of an HIS-tagged TGF-β1 (HIS.TGF-β1) ligand on the cell surface by using an alkaline phosphatase (AP)-conjugated anti-HIS antibody as a secondary detection molecule (see Fig. 6d). Overexpressed HIS.TGF-β1 protein or empty vector-transfected cell-conditioned medium was added to control or FUT8-KO MDA-MB-231 cells endogenously expressing TGF-β receptors. The bound HIS.TGF-β1 protein was quantified by using an AP-conjugated anti-HIS antibody. As compared with the vector control, HIS.TGF-β1 protein showed significant binding on the parental MDA-MB-231 cells as a control, whereas FUT8 knockout markedly decreased TGF-β1 binding on FUT8-KO MDA-MB-231 cells (Fig. 6e). Together, these data suggest that FUT8-modified core fucosylation of TGF-β receptors appears to be critical for their ligand binding and downstream signaling in aggressive breast carcinoma MDA-MB-231 cells.

### FUT8 knockdown reduces lung metastasis of xenograft 4T1 breast tumors

**Fig. 7:**
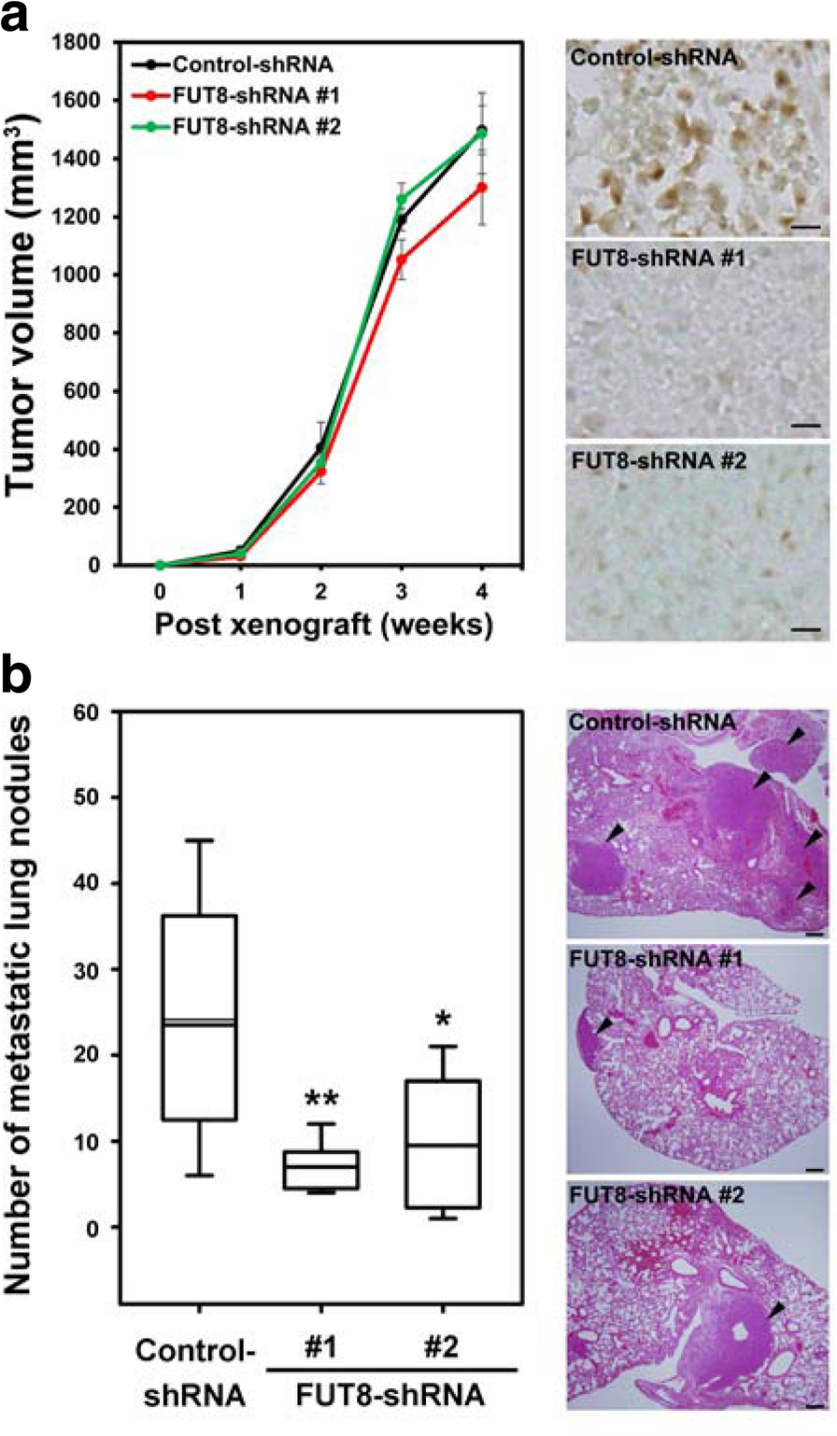
Fucosyltransferase 8 (FUT8) knockdown inhibited the metastatic ability of breast cancer cells in vivo. **a** FUT8 knockdown did not affect xenograft tumor growth of breast cancer cells in vivo. Tumor volumes were measured weekly in nude mice mammary fat pads injected with 4T1 control-short hairpin RNA (shRNA) cells or two independent FUT8-shRNA (#1 and #2) stable cell lines. Data are mean ± SD (n = 8 in each group). Right panel shows representative images of FUT8 expression (brown) in tumor sections from each group. Scale bar = 10 μm. **b** FUT8 knockdown reduced the lung metastasis of the 4T1 tumor model. Number of lung metastatic nodules in nude mice with mammary fat pad injection of 4T1 control-shRNA cells or FUT8-shRNA stable cell lines was counted and is displayed by box-whisker plot (left). Data are median (horizontal line), upper and lower quartiles (box edges) and ranges (whiskers) (n = 8 in each group). *P < 0.05; **P < 0.01 compared with control-shRNA. Representative images of H&E-stained lung sections from each group are shown (right). Arrowheads mark metastatic nodules in the lung. Scale bar = 200 μm

Because FUT8 knockdown inhibited highly invasive 4T1 breast cancer cell invasion in vitro, we next examined whether it could suppress breast tumor metastasis in vivo. Control or FUT8-knockdown 4T1 cells were injected into the mammary fat pads of nude mice, and the number of metastatic nodules in lungs was counted after 4 weeks. FUT8 knockdown did not affect the breast tumor growth at primary sites (Fig. 7a) but greatly reduced the lung metastatic ability of 4T1 cells as compared with the control (Fig. 7b). These results suggest that FUT8 is critically involved in breast cancer metastasis in vivo.

### Pharmacological FUT inhibition suppresses cell invasion and metastasis of breast carcinoma cells

**Fig. 8:**
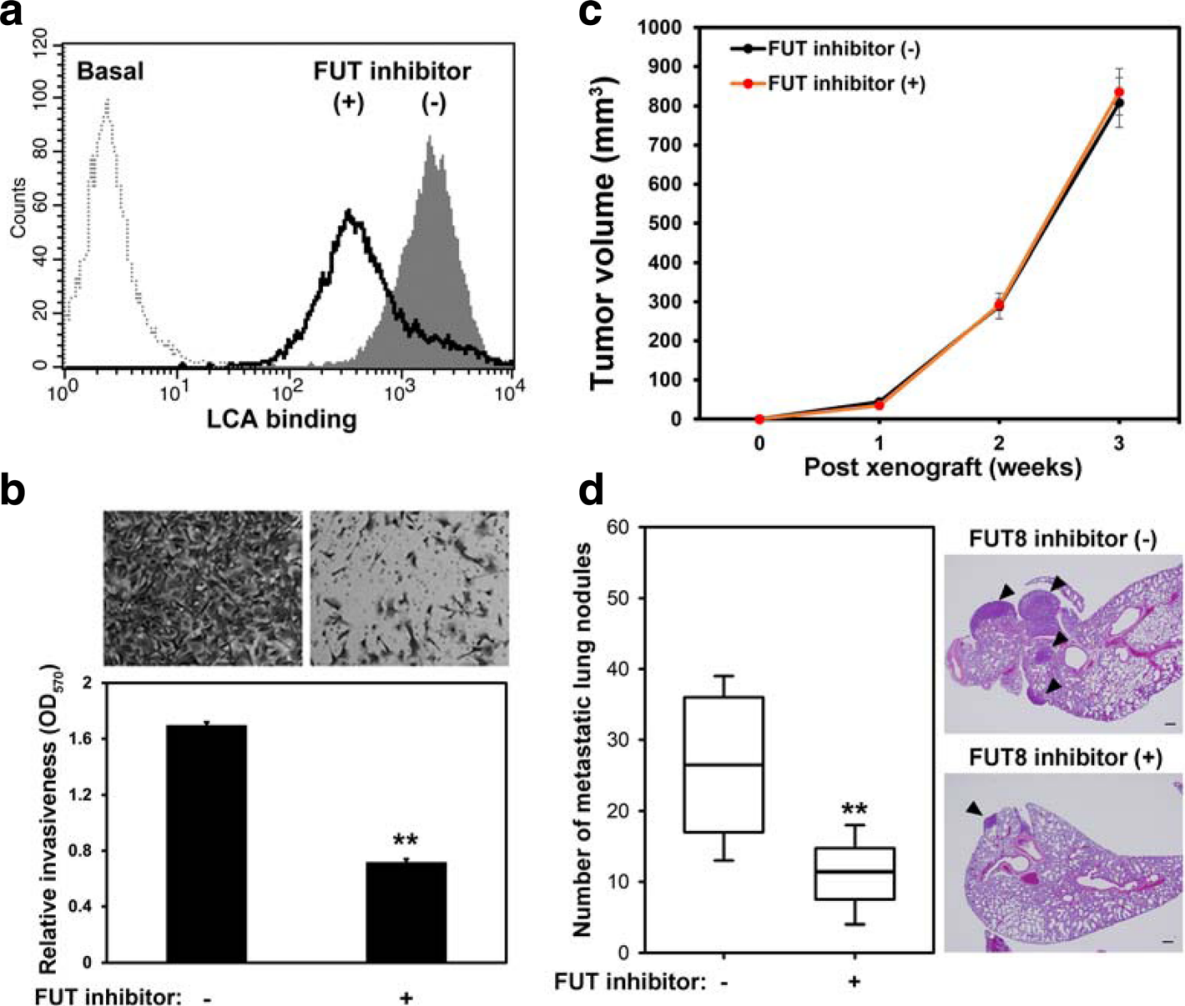
Pharmacological fucosyltransferase (FUT) inhibition reduces invasive ability and lung metastasis of breast carcinoma cells. **a** Core fucosylation was suppressed by treatment with a FUT inhibitor, 2-fluorinated-peracetyl-fucose. MDA-MB-231 cells were treated with 400 μM 2-fluorinated-peracetyl-fucose for 5 days. Core fucosylation of cells was examined by fluorescence-activated cell sorting (FACS) with *Lens culinaris* lectin (LCA) binding assay. **b** Cell invasion measured by the Boyden-chamber Transwell assay with Matrigel coating. Data are mean ± SD. **P < 0.01. **c** FUT inhibitor treatment did not affect xenograft tumor growth of 4T1 cells in vivo. Tumor volumes were measured weekly in nude mice mammary fat pads injected with 4T1 cells. Data are mean ± SD (n = 8 in each group). **d** FUT inhibitor treatment inhibited the lung metastasis of the 4T1 tumor model. Number of lung metastatic nodules in nude mice with mammary fat pad injection of 4T1 cells was counted and displayed by box-whisker plot (left). Data are median (horizontal line), upper and lower quartiles (box edges) and ranges (whiskers) (n = 8 in each group). **P < 0.01 compared with control. Representative images of H&E-stained lung sections from each group are shown (right). Arrowheads mark metastatic nodules in the lung. Scale bar = 200 μm

Our genetic studies clearly demonstrated that FUT8 plays an essential role in breast cancer invasion and metastasis. As a proof-of-concept experiment to test whether FUT8 could be a potential metastatic target, we used pharmacological inhibition with a general fucosylation inhibitor, 2-fluorinated-peracetyl-fucose [20]. MDA-MB-231 cells were treated with this FUT inhibitor for 5 days, and inhibition of core fucosylation was validated by LCA binding (Fig. 8a). In line with genetic inactivation, FUT-inhibitor treatment significantly repressed the invasive ability of MDA-MB-231 cells (Fig. 8b). The effect of the pharmacological inhibitor on lung metastasis of 4T1 cells was further determined in vivo. As shown in Fig. 8c and d, oral administration of a FUT inhibitor (2-fluorinated-peracetyl-fucose) did not affect the primary breast tumor growth but greatly reduced the lung metastatic potential of 4T1 cells as compared with the vehicle control.

## Discussion

**Fig. 9:**
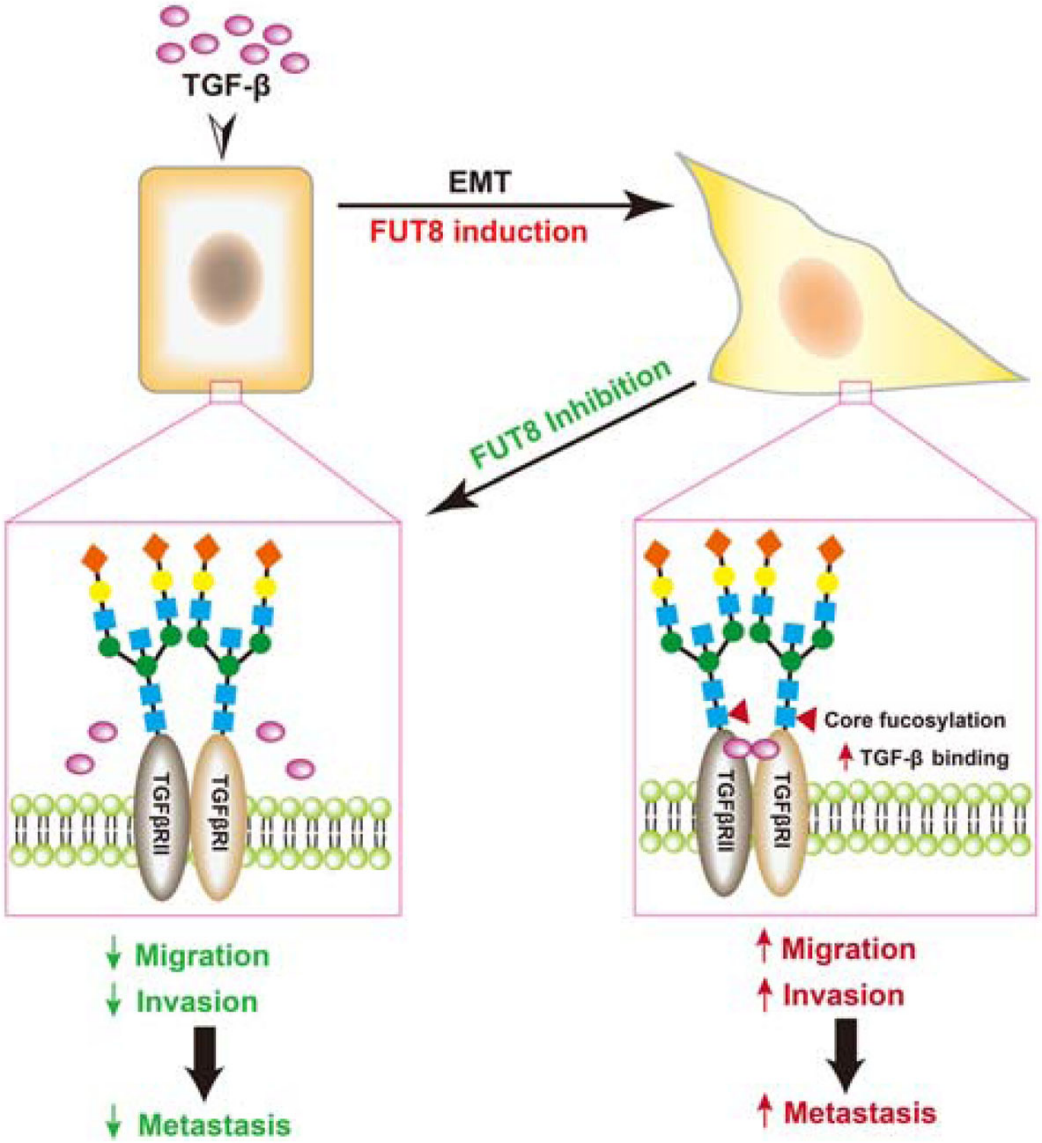
Proposed function of fucosyltransferase 8 (FUT8) in breast cancer progression. Our genetic and pharmacological studies demonstrate that FUT8 is upregulated during epithelial–mesenchymal transition (EMT) of breast cancer cells, and core fucosylation of transforming growth factor-β (TGF-β) receptors enhances ligand binding and promotes downstream signaling activity, for a highly invasive and aggressive phenotype in metastasis of breast cancer cells. Genetic inactivation and pharmacologic inhibition of FUT8 dampens TGF-β receptor core fucosylation, thereby leading to reduced ligand binding and signal strength, which suppresses EMT and therefore cell migration, invasion, and metastasis of breast cancer cells, pointing to FUT8 as a vital target for breast tumors

In this study, we identified a novel positive feedback mechanism for TGF-β signaling associated with breast cancer EMT. FUT8 was upregulated during TGF-β-induced EMT in breast carcinoma cells and upregulated FUT8 remodeled the core-fucosylated N-glycans on cell-surface targets such as TGF-β RI and RII complexes to enhance ligand binding and promote downstream signal activity. Together, these events facilitate the transformation of the epithelial phenotypes toward mesenchymal features, with increased migratory and invasive capabilities in breast cancer cells, potentially leading to distal lung metastasis. Genetic or pharmacologic interruption of this FUT8-promoted TGF-β signaling loop was sufficient to suppress the mobility, invasiveness, and lung metastasis of aggressive breast carcinoma cells both in vitro and in vivo (see Fig. 9).

Although the precise molecular mechanism underlying the upregulation of FUT8 during TGF-β-induced EMT remains elusive in breast carcinoma cells, FUT8 may be modulated in part at the transcriptional level because its mRNA level is highly induced in TGF-β-induced EMT. In agreement with this notion, multiple β-catenin/lymphoid enhancer-binding factor-1 (LEF-1) binding motifs were identified at the 5’ upstream promoter region of FUT8 by *in silico* promoter analysis [32]. Furthermore, downregulation of E-cadherin and nuclear translocation or activation of β-catenin/LEF-1 [33], a major signaling event occurring in EMT, was found associated with FUT8 upregulation during non-small cell lung cancer progression [34]. To determine whether the β-catenin/LEF-1 pathway indeed participates in regulating FUT8 expression in breast cancer cells, we examined the effect of shRNA-mediated β-catenin knockdown on the expression of FUT8 in MDA-MB-231 cells. However, knockdown of β-catenin did not affect FUT8 expression, so unlike in lung cancer cells, TGF-β-upregulated FUT8 may be independent of β-catenin/LEF-1 transactivation in breast cancer MDA-MB-231 cells (Tu et al., unpublished data).

Alternatively, FUT8 may be regulated by a number of EMT-inducing transcription factors (TFs) such as TWIST, SNAIL, SLUG, ZEB1, or ZEB2 [35] because many of these EMT-inducing TFs are E-box binding proteins [36] and the 5’-flanking promoter region of FUT8 gene contains several E-box motifs. In line with these findings, our preliminary screening with FUT8-promoter reporter assay revealed SNAIL but not TWIST, SLUG, ZEB1 or ZEB2 overexpression sufficient to induce the FUT8 promoter-driven reporter activity in breast epithelial MCF-10A cells (Tu et al., unpublished data). Hence, SNAIL may be a regulator of FUT8 during breast cancer EMT. Nevertheless, more investigations are required to further validate the molecular function of SNAIL in FUT8 upregulation with TGF-β treatment in the context of breast carcinoma cells.

Furthermore, we revealed that TGF-β RI or RII proteins, which contain one or three potential N-linked glycosites, were core fucosylated in aggressive breast carcinoma cells. Although Kim et al. recently showed that two conserved asparagine residues, Asn-70 and 94, are N-glycosylated and are essential for the cell-surface transport of TGF-β RII [27], the precise glycan structures and oligosaccharide compositions of these core fucosylated N-glycans in TGF-β receptor complexes remain undefined and need further determination with a combination of specific chemical, enzymatic, mass spectrometry, and high-performance liquid chromatography methods.

Certain N-glycan structures of a number of glycoproteins appear to contribute to the folding, stability, localization, ligand-receptor, and biological functions of the molecules [37]. Here we found that core fucosylation could play an important role in TGF-β-receptor-mediated biological functions. Although the precise structural and functional basis of core fucosylation on TGF-β receptor complexes remains to be elucidated, we proposed some possible mechanisms. First, core fucosylation may cause the conformational alterations of TGF-β receptors. Several studies have reported that changes in the glycan structures of the immunoglobulin G Fc domain and its receptor FcγRIIIa can affect its conformation and ligand-receptor binding affinity [38–40]. Alternatively, core fucosylation might simply affect the cell-surface transport of TGF-β receptors. For instance, Kim et al. recently reported that genetic inhibition or pharmacological suppression of the N-linked glycosylation of TGF-β RII markedly reduced its cell surface transport, thus hindering the interaction with the TGF-β1 ligand [27]. Regardless, additional studies are needed to further elucidate the molecular basis underlying the structural and functional effects after core fucosylation of TGF-β receptors during the EMT.

Besides TGF-β receptors, epidermal growth factor receptor and integrin α3β1 core fucosylation has been shown to potentiate their ligand binding ability [14, 15] and thus may enhance downstream signal pathways to support tumor growth and metastasis. Therefore, an important aspect we are actively pursuing is to identify other FUT8 target proteins and how core fucosylation regulates a specific glycoprotein to particulate in a particular signaling and phenotype during breast cancer EMT. Further understanding the signaling and biological effects of core fucosylation on these target glycoproteins will help decipher the complex molecular mechanisms of FUT8 in breast cancer progression.

## Conclusions

In summary, upregulated FUT8 during TGF-β-induced EMT may represent a novel, vicious feed-forward loop linking core fucosylation and TGF-β receptor signaling to promote EMT and breast cancer progression. Most importantly, inhibition of FUT8 expression or activity may be sufficient to suppress the mobility, invasiveness, and lung metastasis of breast cancer cells in mouse models, which might shed light on devising therapeutic agents to target breast cancer and likely other cancers.

## Additional files

Additional file 1: **Figure S1.** FUT8 knockdown impaired the EMT in NMuMG cells. (PDF 102 kb)

Additional file 2: **Figure S2.** FUT8 expression in invasive or metastatic breast cancers. (PDF 48 kb)

Additional file 3: **Figure S3.** Upregulation of FUT8 is associated with poor prognosis in breast cancer patients. (PDF 41 kb)

## Abbreviations

3D: Three-dimensional
AP: Alkaline phosphatase
Cas9: CRISPR associated protein 9
cDNA: Complementary DNA
CRISPR: Clustered regularly interspaced short palindromic repeats
DMEM: Dulbecco modified Eagle’s medium
EMT: Epithelial–mesenchymal transition
FBS: Fetal bovine serum
FUTs: Fucosyltransferases
GAPDH: Glyceraldehyde-3-phosphate dehydrogenase
H&E: Hematoxylin and eosin
HRP: Horseradish peroxidase
KO: Knockout
LCA: *Lens culinaris* lectin
LEF-1: Lymphoid enhancer-binding factor- 1
mRNA: Messenger RNA
PBS: Phosphate-buffered saline
RPMI: Roswell Park Memorial Institute medium
shRNA: Short hairpin RNA
TFs: Transcription factors
TGF-β: Transforming growth factor-β
