# Supplementary material for: FUT8 promotes breast cancer cell invasiveness by remodeling TGF-β receptor core fucosylation
Source: Breast Cancer Res. 2017 Oct 5;19:111. doi: 10.1186/s13058-017-0904-8 (PMC5629780; doi:10.1186/s13058-017-0904-8)
Supplement: Supplementary file 1 — Figure S1. FUT8 knockdown impaired the EMT in NMuMG cells. (PDF 102 kb) [file 13058_2017_904_MOESM1_ESM.pdf]

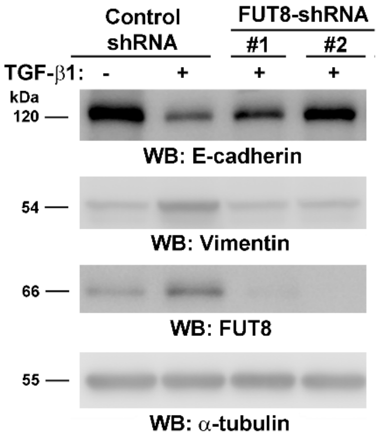

Figure S1. FUT8 knockdown impaired the EMT in NMuMG cells. Control or FUT8-knockdown NMuMG cells were treated with TGF-β1 (5 ng/ml) for 2 days. The effect of FUT8 knockdown on the EMT was examined by measuring E-cadherin and vimentin expression by western blot analysis as previously described.
