# Supplementary material for: FUT8 promotes breast cancer cell invasiveness by remodeling TGF-β receptor core fucosylation
Source: Breast Cancer Res. 2017 Oct 5;19:111. doi: 10.1186/s13058-017-0904-8 (PMC5629780; doi:10.1186/s13058-017-0904-8)
Supplement: Supplementary file 2 — Figure S2. FUT8 expression in invasive or metastatic breast cancers. (PDF 48 kb) [file 13058_2017_904_MOESM2_ESM.pdf]

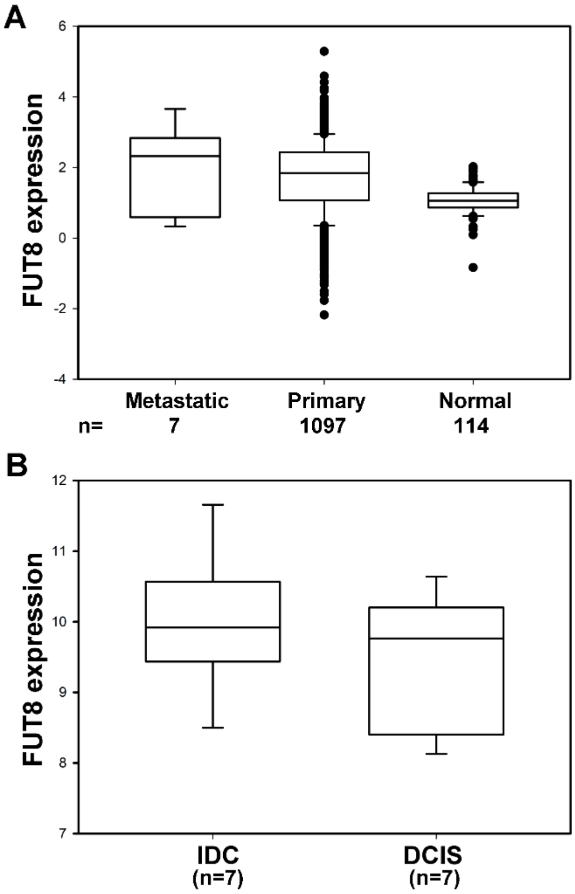

Figure S2. FUT8 expression in invasive or metastatic breast cancers. (A) FUT8 expression analysis of TCGA Breast Cancer (BRCA) cohort using UCSC Xena browser (https://xenabrowser.net). (B) FUT8 expression of matched ductal carcinomas in situ (DCIS) and invasive component (IDC) of seven breast ductal carcinoma patients [1]. Data are median (horizontal line), upper and lower quartiles (box edges) and ranges (whiskers).

Reference

[1] C.S. Schuetz, M. Bonin, S.E. Clare, K. Nieselt, K. Sotlar, M. Walter, T. Fehm, E. Solomayer, O. Riess, D. Wallwiener, R. Kurek, H.J. Neubauer, Progression-Specific Genes Identified by Expression Profiling of Matched Ductal Carcinomas *In situ* and Invasive Breast Tumors, Combining Laser Capture Microdissection and Oligonucleotide Microarray Analysis, Cancer Res., 66 (2006) 5278-5286.
