# Supplementary material for: FUT8 promotes breast cancer cell invasiveness by remodeling TGF-β receptor core fucosylation
Source: Breast Cancer Res. 2017 Oct 5;19:111. doi: 10.1186/s13058-017-0904-8 (PMC5629780; doi:10.1186/s13058-017-0904-8)
Supplement: Supplementary file 3 — Figure S3. Upregulation of FUT8 is associated with poor prognosis in breast cancer patients. (PDF 41 kb) [file 13058_2017_904_MOESM3_ESM.pdf]

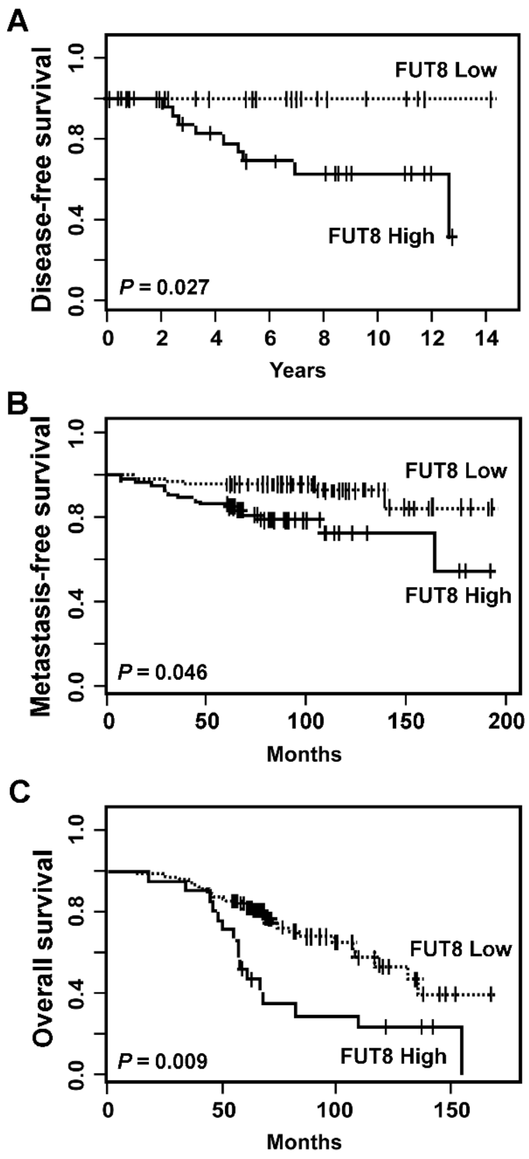

Figure S3. Upregulation of FUT8 is associated with poor prognosis in breast cancer patients. (A) Kaplan-Meier disease-free survival curves for patients with breast cancer by FUT8-high (n= 29) or FUT8-low (n= 25) status [1]. (B) Kaplan-Meier distant metastasis-free survival curves for patients with breast cancer by FUT8-high (n= 67) or FUT8-low (n= 69) status [2]. (C) Kaplan-Meier overall survival curve for patients with breast cancer by FUT8-high (n= 21) or FUT8-low (n= 134) status [3].

References

[1] Y. Zhou, C. Yau, J.W. Gray, K. Chew, S.H. Dairkee, D.H. Moore, U. Eppenberger, S. Eppenberger-Castori, C.C. Benz, Enhanced NF kappa B and AP-1 transcriptional activity associated with antiestrogen resistant breast cancer, BMC Cancer, 7 (2007) 59.

[2] Y. Zhang, A.M. Sieuwerts, M. McGreevy, G. Casey, T. Cufer, A. Paradiso, N. Harbeck, P.N. Span, D.G. Hicks, J. Crowe, R.R. Tubbs, G.T. Budd, J. Lyons, F.C. Sweep, M. Schmitt, F. Schittulli, R. Golouh, D. Talantov, Y. Wang, J.A. Foekens, The 76-gene signature defines high-risk patients that benefit from adjuvant tamoxifen therapy, Breast Cancer Res. Treat., 116 (2009) 303-309.
[3] M. Chanrion, V. Negre, H. Fontaine, N. Salvetat, F. Bibeau, G.M. Grogan, L. Mauriac, D. Katsaros, F. Molina, C. Theillet, J.-M. Darbon, A Gene Expression Signature that Can Predict the Recurrence of Tamoxifen-Treated Primary Breast Cancer, Clin. Cancer Res., 14 (2008) 1744-1752.
